# Immunopathology of Parasitic Infections and Therapeutic Approaches in Humans and Animals

**DOI:** 10.1155/2016/8213532

**Published:** 2016-08-31

**Authors:** Orlando Paciello, Chiara Palmieri, Iwona Otrocka-Domagala, Laura Rinaldi, Jorge Morales-Montor, Peter Geldhof

**Affiliations:** ^1^Department of Veterinary Medicine and Animal Production, Unit of Pathology, University of Naples Federico II, Napoli, Italy; ^2^School of Veterinary Science, University of Queensland, Gatton, QLD 4343, Australia; ^3^Department of Pathological Anatomy, Faculty of Veterinary Medicine, Warmia and Mazury University in Olsztyn, Olsztyn, Poland; ^4^Department of Veterinary Medicine and Animal Production, Unit of Parasitology, University of Naples Federico II, Napoli, Italy; ^5^Departamento de Inmunología, Instituto de Investigaciones Biomédicas, Universidad Nacional Autónoma de México, Circuito Escolar S/N, Ciudad Universitaria, 04510 México, Mexico; ^6^Department of Virology, Parasitology and Immunology, Faculty of Veterinary Medicine, Ghent University, Merelbeke, Belgium

Many studies of the immune system in human and animals have been focused on how it protects the body from parasitic and infectious agents [1], while, recently, many researches were oriented on how defects in the immune system can lead to immunopathological disorders, including autoimmune diseases, immunodeficiencies, and hypersensitivity reactions [1].

Many parasitic diseases are characterized by the long-term persistence of parasites in the host, due to the not fully effective host immunity or inadequate therapy. These conditions have created a particular and somewhat complicated host-parasite relationship supported by mechanisms of immune evasion by parasites, such as parasites covered by self-substances of the host, antigenic variations, and others [2].

Moreover, the first contact between the host and the parasite stimulates the innate immunity, the first immunological, nonspecific mechanism for fighting against infections. This immune response is rapid and is mediated by numerous cells including phagocytes, T-cells, mast cells, basophils, and eosinophils, as well as the complement system [1].

However the infection is established when innate immunity failed in eliminating parasitic and infectious agents and in that case adaptive immunity develops. The primary functions of the adaptive immune response are the recognition of specific “non-self”-antigens, the generation of immunologic pathways for specific pathogens, and the development of an immunologic memory [3]. The cells of the adaptive immune system include T-cells, which are activated through the interaction with antigen presenting cells (APCs), and B-cells [3].

Immune mechanisms are involved in the pathology of many parasitic infections. The main factors responsible for the tissue injury during parasitic infections are chronicity of the infections, release of parasites or host cells in tissues and within the blood, alteration and destruction of the host tissue, presence of antigenic components shared by the host and the parasite, and relative inefficiency of the host in eliminating the antigens or cross-reacting antibodies. The three main pathogenic mechanisms proposed to explain the role of infectious factors as triggers of autoimmune diseases during parasitic infections are (1) polyclonal B- or T-cell activation, (2) increased molecular mimicry, or (3) immunogenicity of self-antigens secondary to the infection-mediated inflammation [4–7].

Even though both immune mechanisms contribute to the development of immunopathological lesions in parasitic diseases, in the past more attention has been given to the humoral response, especially immune complexes and complement.

Immune complexes (ICs) are formed when antibodies bind to relevant antigens: in parasitic diseases, this can occur at the site of parasite penetration, in the circulation, and within tissues or organs. Normally, during the course of the infection, the excess of ICs formed is removed by specific cells, while in pathological conditions the release of massive amounts of antigens from dying parasites which can substantially influence the ratio between antigen and antibody in favour of an antigen excess leads to high amount of ICs that can be detrimental. Furthermore, failure of the reticuloendothelial system to remove ICs from the blood can be another reason of the excess of circulating ICs [7].

Circulating ICs may localize in the walls of blood vessels or in the organs as described in the kidney during leishmaniasis, malaria, trypanosomiasis, schistosomiasis, and other parasitic infections. The local formation of ICs and related diseases has been also observed in several organs such as heart and skeletal muscle lesions in canine leishmaniasis and others [8].

Increased levels of IgE in the host infected with different parasites are a well-known process and they have been used as helpful diagnostic criteria. Their significance in pathology was usually attributed to a type I hypersensitivity reaction, while more recent data have shown that the biological importance of IgE includes other mechanisms [1].

Cell-mediated mechanisms of immunopathological lesions in parasitic diseases are mainly represented by delayed hypersensitivity reactions. A typical example is the granulomatous reaction around eggs or parasites entrapped in the liver, lung, gut, or intestine [9].

It is evident that immune-reactive cells including T-cells, B-cells, and macrophages play important roles in the pathogenesis of these immune-mediated diseases [10]. Recently, it has been shown that several cytokines are responsible for the development and progression of these diseases.

One example is interleukin- (IL-) 6 that is considered one of the major proinflammatory cytokines. It acts on a variety of cells, including immune-competent cells and hematopoietic cells, causing their proliferation and differentiation [11]. The overproduction of IL-6 may be associated with many clinical symptoms observed in inflammatory immune-mediated diseases such as during leishmaniasis [12].

Based on these findings, IL-6 was thought to be a valuable and attractive therapeutic target for drug discovery and some research groups have developed anti-IL-6 receptor antibodies for the treatment of patients with inflammatory autoimmune diseases.

Based on these considerations, much attention has to be given to the response of the immune system during parasitic infections considering immune-mediated associated diseases in the host and considering new targets for combined therapeutic approaches in such conditions.

*Orlando Paciello* *Chiara Palmieri* *Iwona Otrocka-Domagala* *Laura Rinaldi* *Jorge Morales-Montor* *Peter Geldhof*
